# Supplementary figures and images for: TMX5/TXNDC15, a natural trapping mutant of the PDI family is a client of the proteostatic factor ERp44
Source: Life Sci Alliance. 2024 Sep 30;7(12):e202403047. doi: 10.26508/lsa.202403047 (PMC11443168; doi:10.26508/lsa.202403047)

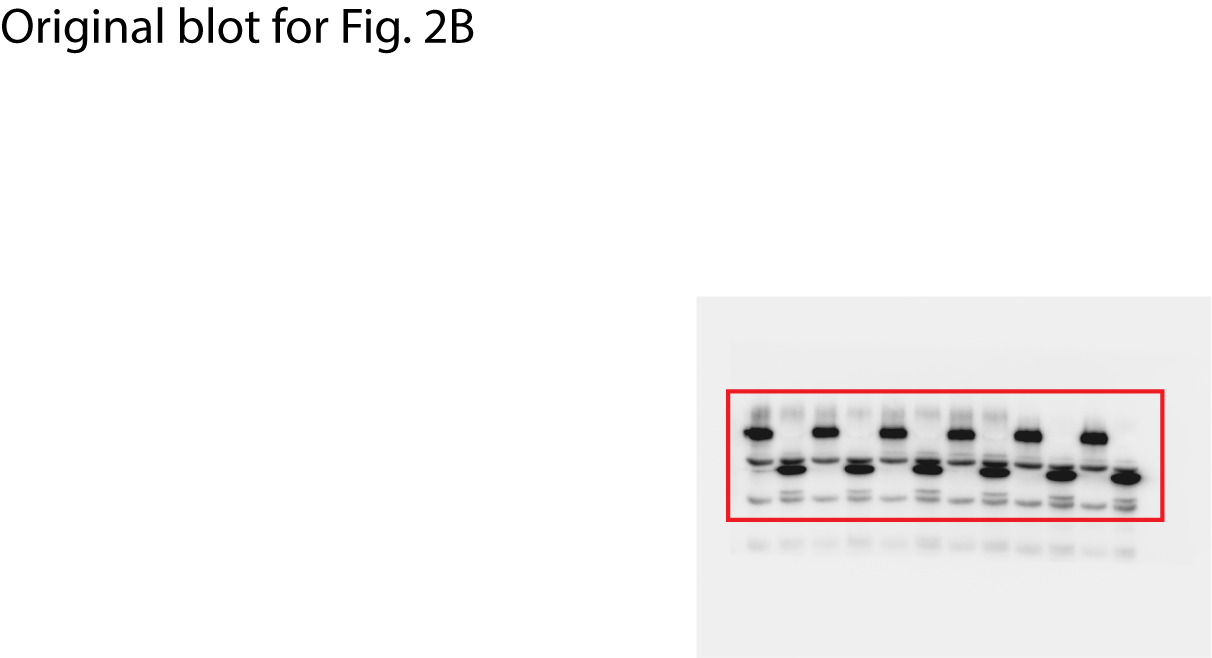

Supplement: Supplementary file 1 [file LSA-2024-03047_SdataF2.tif]
