# Supplementary material for: TMX5/TXNDC15, a natural trapping mutant of the PDI family is a client of the proteostatic factor ERp44
Source: Life Sci Alliance. 2024 Sep 30;7(12):e202403047. doi: 10.26508/lsa.202403047 (PMC11443168; doi:10.26508/lsa.202403047)
Supplement: Supplementary file 2 [file LSA-2024-03047_TableS1.docx]

**Table S1. Details of antibodies used**

| Antibody | Manufacturer | Catalog Nr | Dilution or Concentration |
| --- | --- | --- | --- |
| Mouse anti-V5 | Thermo Fisher Scientific | R96025 | 1:5000 (IB); 1:100 (CLSM) |
| Rabbit anti-HA | Sigma | H6908 | 1:3000 (IB); 1:100 (CLSM) |
| Anti-V5 Agarose Affinity Gel antibody | Sigma | A7345 |  |
| Rabbit anti-CNX | Kind gift from A. Helenius | - | 1:3000 (IB) |
| Mouse anti-GAPDH | Merck | MAB374 | 1:30000 (IB) |
| Rabbit anti-ERp44 | Kind gift from R. Sitia |  | 1:1000 (IB) |
| Rabbit anti-PDI | Stessgen | SPA890 | 1:1000 (IB) |
| Rabbit anti-ERp57 | Kind gift from G. Hämmerling | - | 1:1000 (IB) |
| Rabbit anti-Giantin | Biolegend | 920342 | 1:100 (CLSM) |
| Protein A HRP-conjugated | Invitrogen | 101023 | 1:20000 (IB) |
| Goat anti-mouse HRP-conj | Southern Biotech | 1031-05 | 1:20000 (IB) |
